# Looking Back to *Amycolatopsis*: History of the Antibiotic Discovery and Future Prospects

**DOI:** 10.3390/antibiotics10101254

**Published:** 2021-10-15

**Authors:** Olga V. Kisil, Tatiana A. Efimenko, Olga V. Efremenkova

**Affiliations:** Gause Institute of New Antibiotics, 119021 Moscow, Russia; olvv@mail.ru (O.V.K.); ovefr@yandex.ru (O.V.E.)

**Keywords:** antibiotics, antimicrobial compounds, genus *Amycolatopsis*, glycopeptide antibiotics, polyene antibiotics, rifamycins

## Abstract

The emergence of antibiotic-resistant pathogenic bacteria in recent decades leads us to an urgent need for the development of new antibacterial agents. The species of the genus *Amycolatopsis* are known as producers of secondary metabolites that are used in medicine and agriculture. The complete genome sequences of the *Amycolatopsis* demonstrate a wide variety of biosynthetic gene clusters, which highlights the potential ability of actinomycetes of this genus to produce new antibiotics. In this review, we summarize information about antibiotics produced by *Amycolatopsis* species. This knowledge demonstrates the prospects for further study of this genus as an enormous source of antibiotics.

## 1. Introduction

The science of antibiotics was formed in the twentieth century. About a hundred years ago, Alexander Fleming described the suppression of bacterial growth in an agar medium under the action of a certain substance released into the environment by a fungus colony growing nearby. This fungus was *Penicillium chrysogenum*, and the first discovered antibiotic was called penicillin. In the 1940s, an active search for natural antimicrobial compounds among representatives of various groups of organisms began. In 1952, Zelman Waxman introduced the term “antibiotics”. By the 1960s, all the major groups of currently known antibiotics had been discovered. Unlike the previous half-century period, during which all the main classes of antibiotics were described, in the twenty-first century the effectiveness of the search for new natural antibiotics has significantly decreased. An additional problem was the emergence of antibiotic-resistant microorganisms. The emergence of antibiotic resistance was a natural biological response to antimicrobial drug use, which created selective pressure that promoted the selection, survival and reproduction of microorganism-resistant strains. The spread of antibiotic-resistant microorganisms reduces the effectiveness of prevention and treatment of infectious and parasitic diseases in humans, animals and plants, leads to an increase in the severity and duration of these diseases, an increase in mortality among the population, and the death of animals and plants. The decline in the effectiveness of existing clinically important antibiotics has motivated researchers to search for new molecules with antimicrobial properties to overcome antimicrobial resistance. The phylum *Actinobacteria* represents one of the most diverse groups of microorganisms recognized within the domain Bacteria. Among the phylum *Actinobacteria*, the genus *Streptomyces* is the source of 70–80% of all secondary metabolites; in addition, the important producers of antibiotics are the *Amycolatopsis*, *Actinoplanes*, *Micromonospora* and *Saccharopolyspora* genera [1]. In our review we focus on antibiotics produced by genus *Amycolatopsis*, including the history of their discovery, the emergence of resistance, and the current state of the new drug discovery problem.

## 2. The History and Genomic Analysis of *Amycolatopsis*

The history of the genus *Amycolatopsis* is closely connected with the history of the discovery of antibiotics. The genus *Amycolatopsis* was previously widely used as one of the most effective sources of producers of secondary metabolites with antibacterial, antifungal, or antiviral properties and continues to be the focus of attention when searching for new drugs today [2,3,4]. In the golden era of antibiotics in the 1950s, vancomycin and related glycopeptides (*Amycolatopsis orientalis*) and rifamycin (*Amycolatopsis mediterranei*) were discovered. In addition to antibiotic production, the importance of *Amycolatopsis* strains in industry and ecology, namely bioremediation (heavy metal immobilization, herbicide, and polymer biodegradation) and bioconversion (wuxistatin and vanillin production) was reported [2,5,6]. Some members of the genus *Amycolatopsis* were initially misidentified as *Streptomyces* or *Nocardia*. Only in 1986 did Lechevalier finally recognize *Amycolatopsis* as a unique genus of nocardioform actinomycetes, that lack mycolic acids but contain meso-diaminopimelic acid, arabinose, and galactose in the peptidoglycan of the cell wall [7]. *A. orientalis* was the first recorded species of this genus. The *Amycolatopsis* strains are widespread and are isolated mainly from soil [8]. In addition, the *Amycolatopsis* strains have been isolated from medieval alum slate mine [9], lichen **[10]**, ocean sediment [11], vegetable matter [12], insects [13,14], clinical sources [15,16], and equine placentas [17]. Only four *Amycolatopsis* species are known to have pathogenic properties [15,16,17].

**Figure 1:**
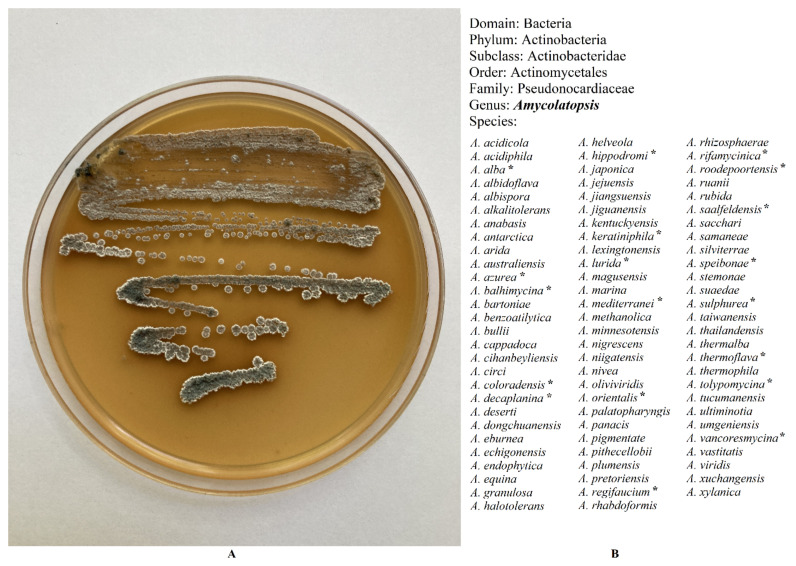
(**A**). *Amycolatopsis orientalis*—vancomycin producer. (**B**). Taxonomy position of the genera *Amycolatopsis*. Nomenclatural status of species: validly published [18]. Note: *—species that are described as antibiotics producers.

As of 2021, the number of officially accepted and published species of the genus *Amycolatopsis* is 83 (Figure 1) [18]. The genome sequences of 120 *Amycolatopsis* strains have been assembled, among them 71 assembled from type material [19]. The genomic studies have revealed that *Amycolatopsis* species have genomes from 5.62 Mb (*A. granulosa* DSM 45669) to 10.94 Mb (*A. anabasis* EGI 650086) (on average, approximately 8.5–9 Mb), circular chromosome, and a high DNA GC content (of 66–75 mol%.) The pan-genome analysis revealed a core genome of 1212 genes with an accessory genome of 27,483 genes and 33,342 unique genes [20,21]. Due to such a significant pan-genome, *Amycolatopsis* species have an extensive adaptive capacity. A major part of the accessory and unique genes of the *Amycolatopsis* strains are involved in secondary metabolite biosynthesis [20,22].

The diversity of secondary metabolites in bacteria is highly dependent on the genus and is mainly organized into several diverse clusters called biosynthetic gene clusters (BGCs), which contain biosynthesis genes in close physical proximity [23,24,25,26]. BGCs encoding for closely related biosynthetic pathways are summarized under the term gene cluster families. All members of the cluster either produce or possess biosynthetic genes for the production of the corresponding class of antibiotics. BGCs of various genera of actinobacteria retrieved from the repository MIBiG (Minimum Information about a Biosynthetic Gene cluster) are presented in Table 1. The correlation between average total genome length and number of BGCs puts *Amycolatopsis* in second place among rare actinobacteria species.

Phylogenetic trees constructed with the oxyB monooxygenase gene (essential for glycopeptides production) or with the AHBA synthase gene (essential for ansamycins production) demonstrate that all strains with correspondent genes are grouped into individual cluster families [27]. So, *Amycolatopsis* type strains that produce, or have the potential to produce, a particular class of antibiotic are phylogenetically related. Owing to this phylogenetic clustering, it is possible to predict the antibiotic-production ability of a novel *Amycolatopsis* strain by its association in the tree, constructed with the antibiotic biosynthetic gene sequences. It should be noted that the presence of these genes does not necessarily mean that the strains will produce the antibiotic. The genes may not be expressed at all in the strain (silent genes) or may only be expressed under specific conditions (e.g., under particular environmental conditions, such as the type of media used for the antibacterial testing). Furthermore, if the genes are expressed, the antibiotic may not have activity against the strains used in the antibacterial screening tests. It is interesting to note that antibiotic biosynthetic genes in several *Amycolatopsis* type strains that are not known to produce antibiotics have been detected.

## 3. *Amycolatopsis* Genomic Potential for Antibiotic Production

Today, understanding of the BGC system functioning, together with next generation sequencing, allows us to predict detection of new antibiotics. In 2006, genomic scanning analyses of *A. orientalis* ATCC 43491, deposited as a vancomycin producer, revealed the presence of genetic loci to produce at least ten secondary metabolites other than vancomycin [28]. Screening of culture liquids led to the isolation of a novel linear polyene antibiotic 13-hydroxy-2,12,14,16,22-pentam ethyl-28-(*N*-methyl-guanidino)octacosa-2,4,6,8,10,14,20,24-octaenoic acid (2-hydroxy-5-oxo-cyclopent-1-enyl)-amide, ECO-0501), which exhibited antibacterial activity against several resistant Gram-positive pathogens.

Bacterial genome sequences are checked for regions that are likely to encode the production of secondary metabolites. Now researchers are faced with another problem: in BGC it is easy to identify bioinformatics, but how do we get them to produce antibiotics in the laboratory? Xu et al. reported a method for activating silent BGCs in diverse microorganisms [29]. This approach relies on elicitor screening to induce the secondary metabolome of a given strain and imaging mass spectrometry to visualize the resulting metabolomes in response to ~500 conditions. Because it does not require challenging genetic, cloning, or culturing procedures, this method can be used with both sequenced and unsequenced bacteria. Application of this method to *Amycolatopsis keratiniphila* NRRL B24117 allowed the discovery of nine glycopeptide chemotype metabolites with potentially therapeutic bioactivities. Keratinimicins A and C showed potent antibacterial activity against numerous Gram-positive pathogens, with minimal inhibitory concentrations (MICs) akin to those of vancomycin against streptococci, *Clostridium difficile*, and *Enterococcus faecalis*.

On the one hand, the detection of ECO-0501 and keratinimicins is a worthy example of using the genome scanning method to identify and isolate a new class of antibacterial preparations. However, on the other hand, genomic mining has not become a key technology for the extraction of natural secondary metabolites. Most natural product BGC identified in bacterial genomic and metagenomic sequencing efforts are silent under laboratory growth conditions. Kim et al. presented a BGC activation method where the gene clusters are disassembled at interoperonic regions in vitro using CRISPR/Cas9 and then reassembled with PCR-amplified, short DNAs carrying synthetic promoters, using transformation-assisted recombination in yeast [30]. This is *in vitro* disassembly/*in vivo* reassembly method was used for the activation of the atolypene BGC from the genome of the cultured actinomycete *Amycolatopsis tolypomycina* NRRL B-24205.30, which led to the characterization of two bacterial cyclic sesterterpenes, atolypene A and B, which are moderately cytotoxic to human cancer cell lines.

The most significant antibiotics from the *Amycolatopsis* genus were isolated by the traditional method, which involves isolation and cultivation of actinobacteria from the soil, screening for inhibitory activity in a test tube, and isolation of the leading molecules. The most currently known antibiotics isolated from *Amycolatopsis* are summarized in Table 2. There are more than 100 compounds of *Amycolatopsis* origin with described antibacterial activity and/or proven antibiotic biosynthesis gene presence. The most productive species are *A. orientalis* (12 antibiotics), *A. mediterranei* (5 antibiotics), and *A. sulphurea* (3 antibiotics). Among the antibiotics produced by *Amycolatopsis*, there are two main commercially significant groups: glycopeptides and polyketides. Further in the text of this review we will discuss this division.

## 4. Glycopeptide Antibiotics

Glycopeptides are glycosylated non-ribosomal peptides produced by a various group of actinomycetes. Glycopeptide antibiotics have a common structure representing a heptapeptide containing aromatic amino acids that have undergone extensive oxidative cross-linking to form macrocycles and carry in various positions such motifs as sugar residues, chlorine atoms, and lipid chains [124]. Among actinobacteria, *A. orientalis* is a well-known producer of glycopeptide antibiotics (Table 2).

**Figure 2:**
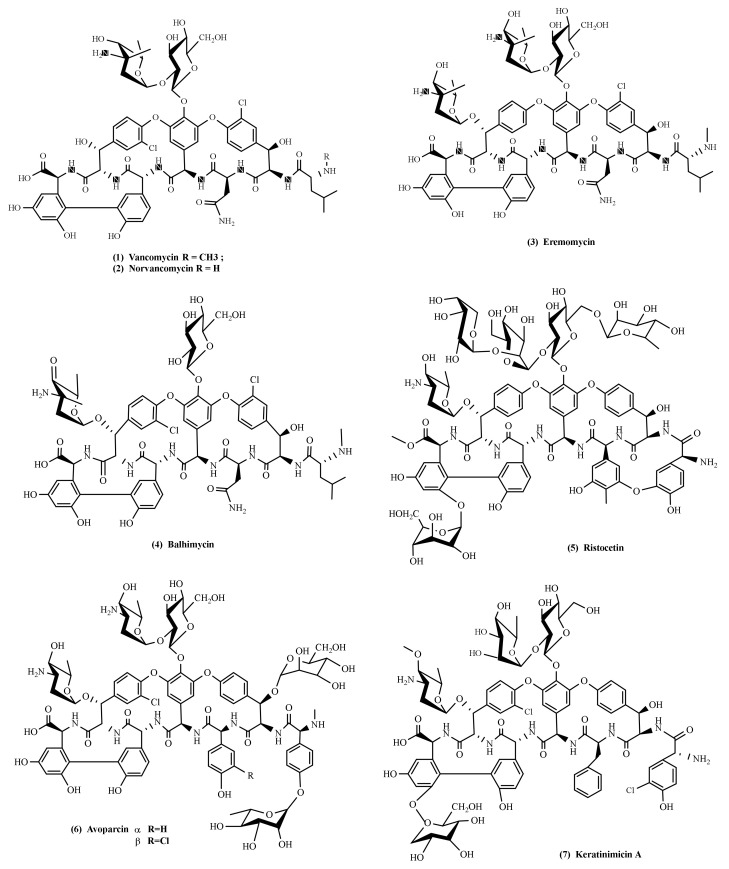
Glycopeptide antibiotics: (**1**) vancomycin, (**2**) norvancomycin, (**3**) eremomycin, (**4**) balhimycin, (**5**) ristocetin, (**6**) avoparcin, and (**7**) keratinimicin A.

Chen et al. proposed the dividing of glycopeptide antibiotics produced by *Amycolatopsis* into three classes, based on residue type at positions 1 and 3 of the heptapeptide: (I) Compounds containing aliphatic residues (vancomycin, balhimycin, eremomycin, chloroeremomycin, orienticin, norvancomycin). Vancomycin and balhimycin contain two sugar residues, while eremomycin and orienticin contain three sugar residues. (II) Compounds containing aromatic residues (avoparcin). (III) Compounds with aromatic residues that are covalently joined to each other (ristocetin) [3]. The structures of the main glycopeptide antibiotics are presented in Figure 2.

### 4.1. Vancomycin

In 1952, a missionary in Borneo sent a soil sample to his friend Dr. E.K. Cornfield, an organic chemist at Eli Lilly and Company [125]. The microorganism isolated from this sample (previously identified as *Streptomyces orientalis*) produced a substance (“compound 05865”) that was active against most Gram-positive organisms, including penicillin-resistant *S. aureus*. Тhe original product, obtained by fermentation, contained considerable (up to 70%) amounts of impurities, and had a brown color, earning it the nickname “Mississippi Mud” [126]. The resulting drug was named “vancomycin”, a term derived from the word “vanquish” [127]. The *A. orientalis* type strain was used for the biological preparation of vancomycin. However, *A. orientalis* is also a producer of natural derivatives of vancomycin, *N*-demethylvancomycin and *N*,*N*-demethylvancomycin, which demonstrate significant antibacterial activity [87,91]. Subsequently, numerous mutant strains of *A. orientalis* were developed for the industrial production of vancomycin, giving a high yield of the drug [128]. In 1958, there was a growing problem of drug-resistant staphylococci, so the US Food and Drug Administration granted vancomycin a “fast track approval” in the absence of an effective alternative [126,129]. However, methicillin, the first semisynthetic penicillin, was also licensed for clinical use in 1958. The pronounced ototoxicity and nephrotoxicity, most likely due to impurities contained in early vancomycin lots, did not allow its widespread use for treatment. A special place among adverse reactions is occupied by the “red man” syndrome, which is characterized by a combination of erythema, pruritis, hypotension, and angioedema. The occurrence of “red man” syndrome is associated with the degranulation of mast cells and basophils caused by the administration of rapid infusions of the first dose of the drug [130]. The aversion to vancomycin is associated with the emergence of methicillin-resistant, and broadly, beta-lactam resistant *S. aureus*, and the introduction of chromatographic purification methods. Chromatographically purified dosage forms of vancomycin with a content of at least 90–95% of the active substance are characterized by low toxicity, and today vancomycin is considered as a relatively safe drug with some minor side effects. Vancomycin and related glycopeptides are considered antibiotics of last resort for the treatment of life-threatening infections caused by all clinically significant Gram-positive human pathogens, such as *Clostridium* spp., *Enterococcus* spp., *Lactobacillus* spp., *Streptococcus pneumoniae*, *S. aureus* (including methicillin-resistant strains of *S. aureus,* MRSA), etc [124,131]. During the vancomycin biosynthesis, seven amino acid precursors are assembled to form a linear heptapeptide, which is then modified, including cyclization, halogenation, methylation, and glycosylation [132,133,134] (Figure S1). Both methylation and demethylation do not affect the antibacterial activity of vancomycin and its derivatives *in vitro*. As for glycosylation, despite aglucovancomycin showing a slightly higher bioactivity than that of vancomycin *in vitro*, the *in vivo* activity was five-fold lower than that of vancomycin [135]. This indicates that part of the sugar may play an important role in giving improved pharmacokinetic properties [135]. Chlorination has not been sufficiently studied, although it is assumed that it improves the dimerization of glycopeptides, which, in turn, can positively enhance antimicrobial activity [133]. The biosynthesis pathways of balhimycin and chloroeremomycin are similar to vancomycin [3].

### 4.2. Eremomycin

Eremomycin was isolated at the Gause Institute of New Antibiotics (Russia) from the cultural liquid of the actinomycete *Nocardia orientalis* INA 238, later clarified as *A. orientalis* [136]. Eremomycin is closely related to vancomycin but differs in sugar residue and chlorine content. Monodechlorovancomycinic acid was detected in eremomycin. The antibacterial spectrum of eremomycin is close to that of ristomycin and vancomycin. However, the *in vitro* antibacterial activity of eremomycin is 2–10 times higher than that of ristomycin and vancomycin. *In vivo* studies showed that eremomycin is less toxic than vancomycin and ristomycin. It does not cause damage to local tissues after intramuscular injections. The chemotherapeutic indices of eremomycin in the treatment of staphylococcal and streptococcal sepsis in albino mice exceeded 10 times those of vancomycin [137]. The pharmacokinetic parameters of eremomycin, teicoplanin, and vancomycin were compared after their intravenous administration to rats at the same dose. The antibacterial activity of eremomycin against methicillin-resistant *S. aureus* (MRSA) was 4 times higher than that of vancomycin [138]. Currently, the ability to produce eremomycin is shown not only for *A. orientalis* but also for *A. umgeniensis* [121].

### 4.3. Norvancomycin

Norvancomycin was isolated from *A. orientalis* CPCC200066 (originally named wan-23) from a soil sample in China in 1959 [93]. This strain was first discovered for its ability to produce an antibiotic that resembles the glycopeptide antibiotic vancomycin, and in 1983 it was confirmed as norvancomycin. The chemical structure of norvancomycin is almost the same as that of vancomycin, except for an absent methyl group at the N-terminus. Norvancomycin is effective for the treatment of bacterial infections caused by Gram-positive cocci and bacilli, especially infections of MRSA and methicillin-resistant *S. epidermidis* (MRSE) [92]. The complete genome sequence of *A. orientalis* CPCC200066 has been obtained [93]. Norvancomycin is widely used in China to treat severe infections such as endocarditis and osteomyelitis.

### 4.4. Balhimycin

Balhimycin was isolated from the fermentation broth of a *Amycolatopsis* sp. Y-86, 21022, later clarified as *A. balhimycina.* It differs from vancomycin only in its glycosylation pattern [55]. Balhimycin is very similar in activity to vancomycin, but it shows higher activity towards anaerobic bacteria. Most knowledge on glycopeptide biosynthetic pathways comes from studies on *A. balhimycina* as this species, among glycopeptide producers, is genetically more amenable [139]. *A. balhimycina* is positioned as a model producing strain for production of improved derivatives of glycopeptide antibiotics by molecular genetic methods [140].

### 4.5. Ristocetin (Ristomycin)

Ristocetin was isolated from *A. orientalis* subsp. *lurida* [41,73]. It was first discovered as a mixture of two closely related components, designated ristocetin A and ristocetin B. Although these two ristocetins have the same antimicrobial spectrum, ristocetin B is 3–4 times more active than ristocetin A. The commercial preparation of this antibiotic is the mixture of ristocetin A and ristocetin B [141]. Ristocetin A and B are specific against Gram-positive bacteria, including mycobacteria. Since the toxic side effects of ristocetin include thrombocytopenia and platelet agglutination, it is only used for laboratory diagnosis of von Willebrand disease. Von Willebrand disease is a mucosal bleeding caused by platelet and collagen binding [142]. One of the strategies to new antibiotics discovery is to evaluate the genetic capacity of the secondary metabolite-producing strains and to activate silent BGC. *A. japonicum* does not produce antibiotics under standard laboratory conditions. To activate a possible silent glycopeptide cluster, Spohn et al. introduced a gene encoding the transcriptional activator of balhimycin biosynthesis, the *bbr* gene from *A. balhimycina* (*bbr*_Aba_), into *A. japonicum*. The resulting recombinant strain of *A. japonicum*/pRM4-*bbr*_Aba_ synthesizes ristomycin A [62].

### 4.6. Avoparcin and Emergence of Vancomycin Resistance

Avoparcin (avotan) was isolated from *A. coloradensis* (formerly *Streptomyces candidus*) in 1968 [56]. It is chemically similar to vancomycin and is a mixture of components. The commercial product consists of a mixture of α- and β-avoparcin, which differ only in the presence of an additional aromatic chlorine atom in the β component [143,144]. Avoparcin has been widely used as a feed additive to promote the growth of cattle, pigs, and chickens. The presence of vancomycin-resistant bacterial strains in humans who were first admitted to the hospital and had never previously taken antibiotics suggested that these strains could have been transmitted through the food chain, as a result of the use of avaporcin in animal feed. The presence of various strains of vancomycin-resistant enterococci strains in animal and human feces in areas where avoparcin was used has been well documented [145]. So, avoparcin was banned in Europe in 1997 by the Commission of the European Union, after which many researchers reported decreased prevalence of vancomycin-resistant enterococci strains in livestock. However, these strains never completely disappeared [146,147].

Antimicrobial activity of glycopeptides is based on binding to the bacterial cell envelope, and not to the target protein, as in the case of most antibiotics. Glycopeptides bind to the *D*-alanyl-*D*-alanine (*D*-Ala-*D*-Ala) dipeptide terminus of the growing peptidoglycan on the outer surface of the bacterial cytoplasmic membrane [148]. This, in turn, interferes with the maturation of the peptidoglycan layer, sequestering the substrate from transpeptidation and/or transglycosylation reactions at the late extracellular stages of peptidoglycan cross-linking. Subsequently, the replicating bacteria cannot survive due to an incomplete and damaged cell wall, which makes them vulnerable to osmotic pressure [124]. Due to a different cell wall morphology, namely the presence of an external lipopolysaccharide membrane impervious to large biomolecules, Gram-negative bacteria are protected from vancomycin [149]. Glycopeptide-resistant organisms replace the *D*-Ala-*D*-Ala terminus with *D*-alanyl-*D*-lactate (*D*-Ala-*D*-Lac) or *D*-alanyl-*D*-serine (*D*-Ala-*D*-Ser), thus markedly reducing antibiotic affinity for the cellular target [150]. Resistance manifests itself in enterococci and staphylococci mainly through the expression of *van* genes encoding proteins that reprogram cell wall biosynthesis and thus evade the action of the antibiotic [124].

The emergence of vancomycin resistance was compared to all other antibiotics. In 1986, vancomycin-resistant *Enterococcus faecium* was found in England and France, followed by vancomycin-resistant *E. faecalis* detected in the United States next year [151]. Vancomycin-resistant enterococci (VRE) are categorized as opportunistic pathogens that are selected for when other bacteria die off. The determinants of resistance in enterococci are encoded in the plasmid-borne transposons, which increases the vancomycin resistance spreading among Gram-positive species through horizontal gene transfer [152,153]. The transfer of *van* genes from enterococci to other Gram-positive bacteria, such as staphylococci, has been shown [154]. The first case of *S. aureus* resistance to vancomycin was detected in 2002 for a dialysis patient in Michigan co-infected with the vancomycin-resistant *E. faecalis* [129,155]. The origin of the genes associated with vancomycin-resistance in enterococci is unknown, but the selection pressure on bacteria was clearly favorable for their occurrence. At the same time, the use of avoparcin in livestock farming has created a hospitable environment for the emergence of vancomycin-resistant enterococci strains. However, it is possible that actinomycetes are the original source of the *van* genes. Most antibiotic-producing bacteria have self-defense strategies and immunity from the effects of these chemical weapons. The simultaneous presence of the antibiotic synthesis and antibiotic resistance genes makes it possible to regulate the bacterial self-resistance [156]. It has been hypothesized that the enterococci vancomycin-resistance genes originated from glycopeptide-producing organisms where they are presumably needed to avoid bacterial suicide [157,158]. Then, the resistance genes were transferred to organisms with the same GC content (for example, *Paenibacillus popilliae*), and then to enterococci. In support of this hypothesis, van-like genes which have similarity to *vanA* and *vanB* have been found in several glycopeptide producers such as *A. orientalis* and *A. balhimycina*.

## 5. Polyketide Antibiotics

**Figure 3:**
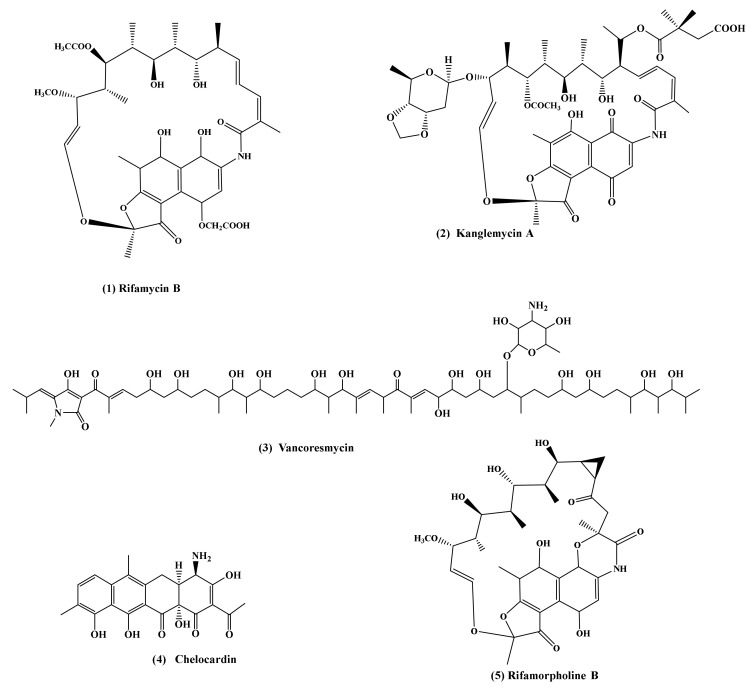
Polyketide antibiotics: (**1**) rifamycin B, (**2**) kanglemycin A, (**3**) vancoresmycin, (**4**) chelocardin, (**5**) rifamorpholine B.

In addition to glycopeptide antibiotics, the genus *Amycolatopsis* is a well-known producer of polyketide antibiotics. Their structures range widely and include cyclic, acyclic, small, large, simple, and complex molecules (Figure 3). Among the polyketide antibiotics produced by genus *Amycolatopsis*, rifamycins, chelocardin, tolypomycin, kanglemicin A, macrothermycins A-D, vanсoresmycin, tetracenomycin X, and rifamorpholines A-E should be listed (Table 2). These antibiotics are united by their bacterial biosynthetic pathway: all of them are obtained through a polyketide precursor, which is different in the case of each antibiotic. The most commercially demanded of them is rifamycin, which belongs to the ansamycin polyketides. Ansamycins get their name from the characteristic configuration of their molecule carbon skeleton, which has a basket-shaped architecture, consisting of an aromatic naphthalene (or benzene) core and a long aliphatic bridge in the shape of a handle (latin, *ansa*) connecting two non-adjacent positions of the core. The resulting molecules are very rigid and compact, which leads to unique chemical properties and specific biological effects [159].

### 5.1. Rifamycin’s Discovery and Structure

In 1957, in France, from a soil sample in Saint-Raphael, a strain was isolated which was classified as *Streptomyces mediterranei*, later as *Nocardia mediterranei*, and finally as *Amycolatopsis mediterranei* ATCC 13685/DSM 43304/ME 83/973. The strain was cultured in shaking flasks and the cultural liquid showed high activity against Gram-positive bacteria *Mycobacterium tuberculosis*. In addition, it demonstrated limited activity against some Gram-negative bacteria [79]. Thus, one of the earliest antibiotics was discovered, rifamycin, named after the Italian movie “Le Riffi” [160]. The original strain *A. mediterranei* ATCC 13685 produced a mixture of several rifamycin antibiotics. The only component of this extract that could be isolated in pure crystalline form by the addition of sodium diethylbarbiturate was rifamycin B, secondary in biological activity. The rifamycin B molecule consists of two main parts: the naphthoquinone ring and a 24-member aliphatic chain with 5 methyl groups (Figure 3(**1**)). Due to the importance of rifamycin, the producer strain *A. mediterranei* was selected in order to create strains capable of producing large amounts of rifamycin B, or its biologically active natural derivatives [161]. Later, a mutant strain, *A. mediterranei* ATCC 21789, producing single rifamycin B without any barbiturate salt addition, was isolated [162].

All ansamycins are assembled by the polyketide pathway, using 3-amino-5-hydroxybenzoic acid (AHBA) as the starting unit [163]. The earliest macrocyclic precursor in the biosynthesis of rifamycin is proansamycin X (Figure S2). It had never been isolated and identified, therefore, it is to some extent hypothetical [163,164]. Proansamycin X dehydrogenation leads to the formation of biologically inactive rifamycin W. Further post-translational modifications lead to the production rifamycin SV, and rifamycin S [165]. Rifamycins W, S, and SV are key intermediates in biosynthesis and are precursors of many other natural derivatives of rifamycins: B, R, G, Q, P, Z, O, L, Y, etc. Table 3 summarizes the rifamycin derivatives produced by genus *Amycolatopsis* and shows their bioactivity and biosynthetic precursors. Some of these derivatives, together with ketides accumulated by *A. mediterranei*, can be considered as waste metabolites, resulting from enzymatic reactions with the formation of biologically active rifamycins. The most stable component in the rifamycin complex is rifamycin B. Reversible oxidation of the quinone core, followed by hydrolytic loss of the glycolic acid fragment of rifamycin B, leads to the production of significantly more active rifamycins S and SV. Rifamycin SV quickly stood out among the first available natural rifamycins due to its antibacterial activity and low toxicity. Rifamycin SV was the first rifamycin used in clinical practice but was only effective when injected intravenously [166]. Rifamycin S was half as weak as rifamycin SV [167]. The poor bioavailability and poor pharmacokinetic properties of rifamycin SV, combined with the understanding of structure–activity relationships, initiated a chemical campaign to develop a more potent and orally bioavailable drug. In 1965, Dow-Lepetit Research Laboratories (Milan, Italy) developed *rifampicin* (3-(4-methyl-piperazinyl-iminomethyl) rifamycin SV), which is the most important and widely used semi-synthetic antibiotic of the rifamycin group in medicine (Figure S2) [168].

### 5.2. Mechanism of Rifampicin Action and Occurrence of Resistance

The antibiotic activity of rifamycins on the bacterial cell has been most widely studied for rifampicin. Rifampicin (and other rifamycins) binds in the rifamycin-binding pocket to the β-subunit of RNA polymerase in the immediate vicinity of the catalytic site and sterically blocks the expansion of the RNA chain [167]. The collision of RNA and rifampicin occurs when the RNA reaches a length of 3–4 nucleotides, after which the RNA is released from the promoter complex in the form of an interrupted transcript. The length of the abortive RNA product may be affected at the C3 substituent of a particular rifamycin’s derivative. Eukaryotic cell polymerases are less sensitive to the antibiotic compared to the bacterial ones. Binding constants for prokaryotic RNA polymerases are about 10^−8^ M whereas those for eukaryotic enzymes are at least 10,000 fold weaker [167]. Due to their high selectivity for their molecular target, rifamycins have become a safe and effective drug [78]. At present, rifampicin is still the first-line treatment for diseases such as tuberculosis, leprosy, and various infections associated with the biofilm formation. It is important to note that natural rifamycins have significant activity only against Gram-positive bacteria because of the hydrophobic nature of their large molecule.

The main practical rifamycin application is associated with its activity against mycobacteria. Tuberculosis is second (just after AIDS) among the world’s most common causes of death from infectious diseases [188]. WHO estimates that 8–10 million new cases of tuberculosis occur worldwide each year. A third of the world’s population is infected by *M. tuberculosis*, the etiological agent of tuberculosis [189,190]. However, long periods of use and poor medical supervision have resulted in rifamycin-resistant *M. tuberculosis* strains [191]. The primary mechanism of resistance to rifampicin (and other rifamycins) consists of rapid selection of resistant mutants (amino acids substitutions) in the rifampicin-binding pocket of RNA polymerase, which results in antibiotic affinity decreasing. Another way to decrease antibiotic affinity is the enzymatic modification of rifamycin by C-21 and C-23 hydroxyl groups [192]. Altogether, these modifications generate the rifamicyn resistome, which negatively affects this class of antibiotics. In 2016, there were 600,000 reported new cases of resistance to rifamycin, of which 490,000 were caused by multidrug-resistant *M. tuberculosis* strains [78,193].

### 5.3. Polyketide Backbone Rearrangement

Despite obtaining a large number of rifamycin derivatives by semi-synthetic approaches (more than 750 rifamycin derivatives have been studied) the possibility of chemically introducing structural modifications is limited because of the structural complexity of the rifamycin molecule [160,169]. Unfortunately, no new drug has been developed for tuberculosis in recent decades [167]. Today, to achieve structural diversity, researchers have switched to a combinatorial biosynthetic approach—mutasynthesis. Knowledge of the biosynthesis of rifamycins allows the rational genetic manipulation of *A. mediterranei* to obtain new natural antibiotics.

Nigam et al., by replacing the substituted acyltransferase domain of module 6 of rifamycin polyketide synthase with that of module 2 of rapamycin polyketide synthase, obtained the semisynthetic derivatives 24-desmethylrifamycin B and 24-desmethylrifamycin SV. These compounds have proven effective against a number of pathogenic bacteria, including several rifampicin-resistant *M. tuberculosis* strains [194].

Posttranslational modifications at the last stages of the biosynthetic pathway of rifamycins play an important role in expanding the structural diversity and, as a consequence, biological activity of the final rifamycin metabolites. Table 3 shows that the early intermediates on the pathway of rifampicin biosynthesis do not have any biological activity. The proposed earliest macrocyclic precursor in rifamycin biosynthesis, proansamycin X, undergoes dehydration to form protorifamycins or undergoes dehydrogenation to form rifamycin W. Rifamycin W undergoes a rearrangement of the polyketide backbone to produce rifamycin B. However, the progress of genetic engineering allows us to look at inactive rhymaicins X and W as potential sources for structural modifications in the hope of new drugs discovery. Shi et al. in 2021 constructed the mutant strain *Amycolatopsis mediterranei* S699 *Δrif-orf5* by in-frame deleting the *rif-orf5* gene (involved in the polyketide backbone rearrangement mechanism) to afford thirteen rifamycin W congeners including seven new ones [195]. Compounds 1–3 exhibited antibacterial activity against *Staphylococcus aureus*. A year earlier, Ye et al. constructed mutant strain *A. mediterranei* S699*ΔrifT* by deleting the *rifT* gene, encoding NADH-dependent dehydrogenase, presumably responsible for the dehydrogenation of proansamycin X. The mutant strain successfully produced eleven 8-deoxy-rifamycin derivatives and seven known analogs. For four of them, antibacterial activity against *S. aureus* was shown [196].

## 6. Old New Polyenes

A promising approach to searching for effective antibiotics is to look back and re-examine the molecules that previously demonstrated antibacterial activity but for various reasons did not receive further development.

### 6.1. Kanglemycin A

As well as rifamycins, *A. mediterranei* produces another ansamycin—kanglemycin A (KglA). KglA was originally isolated from the fermentation broth of *Nocardia mediterranei* var. *kanglensis* 1741–64 [77]. There was only limited information about its biological activity until 2018, when Mosaei et al. described in detail the mechanism of KglA action [78]. This antibiotic contains two important and unusual ansa bridge modifications: a pendant 2,2-dimethyl succinic acid side chain at C20 and a unique sugar moiety (β-O-3,4-O,O’-methylene digitoxose) at C27. As a result, KglA exhibits an altered binding conformation with RNA polymerase (larger binding surface) in comparison to known rifamycins and their semisynthetic derivatives. The mechanism of KglA action also differs from rifampicin, as KglA inhibits RNA synthesis even after the first phosphodiester bond formation. This leads to the phenomenon where KglA is effective against rifampicin-resistant pathogens [78,197].

### 6.2. Chelocardin (Otherwise Known as Cetocycline or Cetotetrine)

Another polyenes antibiotic produced by *Amycolatopsis* that has regained interest in recent years is atypical tetracycline chelocardin. Isolated from *A. sulphurea* (formerly *N. sulphurea*), chelocardin was first described in the 1970s [116,198]. It is structurally related to tetracyclines and contains a 9-methyl group, aromatic ring, unsubstituted 4-ammonia group, and the methyl group replacing the 2-ammonia group. At low concentrations, like classical tetracyclines, chelocardin prevents bacterial growth by inhibition of peptidyl transferase biosynthesis. At higher concentrations, the bacterial membrane is the main antibiotic target of chelocardin [199]. The application of the biosynthetic engineering approach made it possible to design a recombinant *A. sulphurea* producing a new chelocardin analogue with carboxamido moiety of tetracyclines (an important structural feature for its bioactivity). 2-Carboxamido-2-deacetyl-chelocardin showed significantly improved antimicrobial activity against a collection of well-characterized multidrug-resistant clinical isolates from the ESKAPE panel [118,200,201].

### 6.3. Vancoresmycin

Vancoresmycin is an understudied natural product antibiotic consisting of a terminal tetramic acid moiety linked to a linear, highly oxygenated, stereochemically complex polyketide chain. It was isolated from the fermentation broth of the *Amycolatopsis* sp. ST 101170 in 2002 [122]. The species name *A. vancoresmycina* was proposed by Wink et al. who isolated it from Indian soil [65]. In 2013 the genome of the strain *A. vancoresmycina* DSM 44592 was sequenced [202]. Vancoresmycin shows minimal inhibitory concentrations against a range of clinically relevant, antibiotic-resistant Gram-positive bacteria. It selectively targets the cytoplasmic membrane of Gram-positive bacteria via a concentration-dependent depolarization mechanism [123].

### 6.4. Rifamycin O

Some studies return attention to the natural metabolites of rifampicin, which were not tested in time due to the establishment of rifampicins B, S, and SV for clinical purposes (Table 3). In 2020 it was shown that rifamycin O, which is fundamentally different from other rifamycins in positions C1 and C4, showed significant activity *in vitro* and *in vivo* against *M. abscessus.* It is the most difficult-to-treat nontuberculous mycobacteria because of internal and acquired resistance mechanisms and *M. abscessus* cell wall is 10–20 times less permeable than that of *M. tuberculosis* [183].

## 7. Antibiotics Produced by *Amycolatopsis* Isolated from Poorly Studied Ecological Habitats

While metagenomics and high-throughput sequencing tools reveal the species diversity of microbial communities and identify genetic clusters for the production of antibiotics that have not been detected by cultured approaches, the isolation of a monoculture of microorganisms is still important for the detection of bioactive compounds. However, in order to effectively obtain *Actinobacteria* for the discovery of new drugs, it is necessary to estimate where to search for new producers in terms of geography and specific ecological systems [203]. The main hopes for new antibiotic discoveries are related to microorganisms isolated from extreme or unusual environments that are characterized by challenging conditions such as aridity, high salinity, low nutrient sources, extreme temperatures, and especially the complex composition of microorganism species. Other alternative promising sources of specialized metabolites are the microbiota of diverse eukaryotic hosts, including plants, insects, sponges, and humans. The evolution of microorganisms from such habitats follows a special path, due to geographical and/or genetic isolation and adaptation to extreme conditions. Therefore, it is likely to find among such endemic species unique metabolisms, the products of which are new antibiotics. In the last five years, several new antibiotics produced by various *Amycolatopsis* strains have been isolated and described. Most of them are located in special environmental conditions. Recent studies of lichen-associated *Amycolatopsis* metabolites have led to the isolation of amycophthalazinone A [44], 2-carbamoyl-3-hydroxy-1,4-naphthoquinone [45], and amycolasporin C [61]. Amycophthalazinone A is the first example of a naturally occurring phthalazinone derivative. Amycophthalazinone A exhibits potent inhibitory activity against *S. aureus* and *Salmonella typhi* (MIC 32 μg/mL) [44]. The antimicrobial activity test shows that 2-carbamoyl-3-hydroxy-1,4-naphthoquinone has significant inhibitory effects on bacterial pathogens MRSA (MIC 2 μg/mL) and fungal pathogens of *Botrytis cinerea* and *Fusarium graminearum* (MICs 1 μg/mL) [45]. Amycolasporin C shows activity against *Bacillus subtilis, S. aureus,* and *Escherichia coli* (MIC 25 μg/mL) [61]. The sponge-associated marine bacteria produce more antibiotic substances through competition for space and nutrients. Amycolactam, isolated from the *A. saalfeldensis*, is the bacterial indole alkaloid related to the cyclopiazonic acid class [32]. The biological activities of amycolactam were evaluated in antibacterial and antifungal assays against various pathogenic microbes, but the compound did not exhibit significant inhibitory activities. Amycolactam displays significant cytotoxicity against the gastric cancer cell line and the colon cancer cell line, with IC_50_ values of 0.8 and 2.0 μM, respectively. Bacteria, through antibiotics, often provide chemical defenses that selectively inhibit insect microbial competitors and pathogens. Macrotermycins A and C from the termite-associated *Amycolatopsis* sp. M39 have antibacterial activity against human-pathogenic *S. aureus* (MIC 1.5 and 10 μg/mL, respectively) [14]. *Amycolatopsis* sp. HCa4 isolated from the gut of locusts (*Locusta migratoria*) produces amycolamycin A which is selectively cytotoxic to the M231 breast cancer cell line [204] and rifamorpholines A-E [13]. Rifamorpholines represent the new subclass of rifamycin antibiotics with an unprecedented 5/6/6/6 fused tetracyclic ring system and an unusually modified polyketide chain. Rifamorpholine B (Figure 3) shows activity against MRSA (MIC 4 µg/mL).

## 8. Conclusions

Medical success in the treatment of many diseases is associated with the development and widespread use of antibiotics, biologically active substances of natural origin, and their chemical analogues with antimicrobial, antitumor, antiviral, and immunomodulatory properties. At the beginning of the birth of the science of antibiotics, which began with the discovery of penicillin in 1928, the search for new antibiotics has been carried out in a variety of organisms. Later it was shown that most antibiotics are formed by fungi and bacteria living in species-enriched biocenoses, primarily in the soil. It was found that the main producers of antibiotics are actinobacteria. Actinobacteria produce two-thirds of all known antibiotics used in the clinic today. Among actinobacteria, representatives of the genus *Streptomyces* are the champions in a number of identified antibiotics. Unfortunately, at present, the discovery of new natural antibiotics is not as effective as it was in the “golden era of antibiotics” (1940s–1970s). The study of rare genera of actinobacteria, which are not as thoroughly studied as *Streptomyces*, is promising for the search for new antibiotics. Among such genera, the genus *Amycolatopsis* is particularly interesting, since its representatives form antibiotics of different chemical structures, including two especially important medical antibiotics, vancomycin and rifamycin, and their analogues. The sequencing of the first complete bacterial genome in 1995 opened a new page of possibilities for antibacterial drug discoverers. The combination of next-generation sequencing technologies, comparative genomics, and studies of the role of specific gene expression provides effective opportunities for activating the BGCs that *Amycolatopsis* is so full of. Silent BGCs are a treasure trove of potential new antibiotics. An alternative approach to the search for new antibiotics is to optimize the structural scaffolds with proven antibacterial activity by genetically engineering strains producing commercially significant antibiotics, such as *A. mediterranei* and *A. orientalis*. Transformation of compounds such as rapamycin through the application of biosynthetic engineering can deliver novel drug candidates. Every year, the genus *Amycolatopsis* opens up new prospects for obtaining new antibiotics. In the past five years, more than a dozen new antibiotics produced by strains of various species of *Amycolatopsis* have been isolated and described. The results of our review show that members of the genus *Amycolatopsis* are still a valuable source of new antibiotics, and our task is to correctly reveal and use this potential.

## Figures and Tables

**Table 1:** Number of identified biosynthetic gene clusters for various genera of actinobacteria.

Genus	Average Total Genome Length (Mb) [19]	Number of Biosynthetic Gene Clusters [25]
*Actinoplanes*	9	11
*Actinomadura*	9	10
*Amycolatopsis*	9	25
*Micromonospora*	7	26
*Nocardia*	8	6
*Streptomyces*	9	637
*Streptosporangium*	10	3

**Table 2:** Representatives of the genus *Amycolatopsis* and the antibiotics they produce.

Species, Strains	Antibiotics	Properties	References
*Amycolatopsis* sp. 17128	Mutactimycin A, D, E	Antimicrobial activity against Gram-positive bacteria (including MRSA ^1^)	[31]
*Amycolatopsis* sp. Cra33g	Amycolactam	Significant cytotoxicity	[32]
*Amycolatopsis* sp. Hca4	Rifamorpholines A–E	Antimicrobial activity against Gram-positive bacteria (including MRSA)	[13]
*Amycolatopsis* sp. IRD-009	Pradimicin-IRD	Antimicrobial activity against Gram-positive and Gram-negative bacteria; Cytotoxic activity against cancer cell lines	[33]
*Amycolatopsis* sp. К16-0194	Dipyrimicins A and B	Dipyrimicin A exhibits strong antimicrobial and cytotoxic activities; Dipyrimicin B exhibits antimicrobial activity against *Escherichia coli*	[34]
*Amycolatopsis* sp. LZ149	Siderochelins A, D, E, and F	Siderochelin A exhibits antimicrobial activity against Gram-positive bacteria and *Escherichia coli*; Siderochelins A, D and E exhibit antimicrobial activity against *Mycobacterium smegmatis*	[35,36]
*Amycolatopsis* sp. M39	Macrotermycins A–D	Macrotermycins A and C had antimicrobial activity against Gram-positive bacteria (particularly staphylococcal infections); Selective antifungal activity (against a fungal parasite of the termite fungal garden)	[14]
*Amycolatopsis* sp. MI481-42F4	Аmythiamicins A, B, C and D	Antimicrobial activity against Gram-positive bacteria (including MDR ^2^ strains)	[37,38]
Amycolatopsis sp. MJM2582	Ristocetin (Ristomycin)	Antimicrobial activity against Gram-positive pathogenic infections (particularly staphylococcal infections); Applied to the in vitro diagnosis of conditions such as von Willebrand disease and Bernard–Soulier syndrome	[39,40,41]
*Amycolatopsis* sp. ML1-hF4	Рargamicins A	Antimicrobial activity against *Staphylococcus aureus* strains (including MRSA) and *Enterococcus faecalis, E. faecium* strains (including VRE ^3^)	[42]
	Valgamicins A, C, T and V	Weak activity against Gram-positive and Gram-negative bacteria; Valgamicins A, C and T exhibit moderate cytotoxicity against human tumor cell lines	[43]
*Amycolatopsis* sp. YIM 130642	Amycophthalazinone A	Weak antimicrobial and antifungal activities	[44]
*Amycolatopsis* sp. YIM 130687	2-carbamoyl-3-hydroxy-1,4-naphthoquinone	Strong antimicrobial (including MRSA) and antifungal activities	[45]
*Amycolatopsis* sp. AA4	Amycomicin	Strong antimicrobial activity against *Staphylococcus aureus*	[46]
*A. alba*	1(10-aminodecyl) pyridinium	Antimicrobial activity against Gram-positive and Gram-negative bacteria; Cytotoxic activity against cancer cell lines	[47]
	Kigamicins A-E	Antimicrobial activity against Gram-positive bacteria (including MRSA); Kigamicin D is an anticancer agent	[48,49]
	Maytansinoids 1–14	Maytansinoids 7 and 13 showed antitumor activities against four human cancer cell lines	[50]
*A. australiensis*	Antibiotic biosynthetic genes were identified		[27]
*A. azurea*	Azureomycins A and B	Strong antimicrobial activity against Gram-positive bacteria	[51,52]
	Оctacosamicins A and B	Very weak or no activity against Gram-positive and Gram-negative bacteria; Moderate activity against fungi and yeast	[53,54]
*A. balhimycina*	Balhimycin	Antimicrobial activity against Gram-positive bacteria (including MRSA)	[55]
*A. coloradensis*	Avoparcin (avotan)	Antimicrobial activity against Gram-positive bacteria; Animal growth promoter	[56,57,58]
*A. decaplanina*	Decaplanin	Antimicrobial activity against Gram-positive bacteria (including antibiotic-resistant enterococci and clinical isolates)	[59,60]
*A. hippodromi*	Amycolasporins A−C	Antimicrobial activity against Gram-positive and Gram-negative bacteria	[61]
*A. japonica*	Ristocetin (Ristomycin)	Antimicrobial activity against Gram-positive bacteria, (particularly staphylococcal infections); Applied to the in vitro diagnosis of conditions such as von Willebrand disease and Bernard–Soulier syndrome	[62]
*A. jejuensis*	Antibiotic biosynthetic genes were identified		[63]
*A. keratiniphila*	Keratinimicins A–D; Keratinicyclin A–C	Keratinimicins A and C exibit strong antimicrobial activity against Gram-positive bacteria (particularly staphylococcal infections); keratinicyclin В exibit moderate antimicrobial activity against *Streptococcus* spp. and *Clostridium difficile*	[29]
*A. keratiniphila* subsp. *keratiniphila*	Antibiotic biosynthetic genes were identified		[27]
*A. keratiniphila* subsp. *nogabecina*	Nogabecin (Actinoidin B)	Antimicrobial activity against Gram-positive bacteria	[64,65]
*A. lactamdurans* *	Cephamycin C	Antimicrobial activity against Gram-positive and Gram-negative bacteria (including resistant strains); very efficient antibiotic against anaerobic microbes	[66,67,68,69]
	Efrotomycin	Antimicrobial activity against Gram-positive bacteria	[68,70]
*A. lurida*	Benzanthrins A and B	Antimicrobial activity against Gram-positive bacteria; Inhibit the growth tumor cells in tissue culture	[71,72]
*A. lurida*	Ristocetin (Ristomycin)	Antimicrobial activity against Gram-positive bacteria, (particularly staphylococcal infections); Applied to the in vitro diagnosis of conditions, such as von Willebrand disease and Bernard–Soulier syndrome	[41,73,74]
*A. mediterranei*	Amexanthomycins A–J	Inhibitory activity against human DNA topoisomerases	[75]
	Dethymicin	Antimicrobial activity against Gram-positive bacteria (including MRSA); Immunosuppressant	[76]
	Kanglemycin A	Antimicrobial activity against Gram-positive bacteria (including rifampicin-resistant ones and *M. tuberculosis* with MDR)	[77,78]
	Rifamycines	Strong antimicrobial activity against Gram-positive bacteria (particularly mycobacteria)	[79,80,81,82,83]
	Tetracenomycin Х	Antimicrobial activity against Gram-positive bacteria; Showed antitumour activity *in vivo*	[84,85]
*A. minnesotensis*	Antibiotic biosynthetic genes were identified		[27]
*A. nigrescens*	Antibiotic biosynthetic genes were identified		[27]
*A. niigatensis*	Antibiotic biosynthetic genes were identified		[27]
*A. orientalis*	Vancomycin	A last-line drug for the treatment of infections caused by almost all clinically significant Gram-positive bacteria (including MRSA)	[3,86]
	*N*–Demethylvancomycin	Antimicrobial activity against Gram-positive bacteria (including MRSA)	[87,88,89,90]
	*N*,*N*–Demethylvancomycin	Antimicrobial activity against Gram-positive bacteria	[91]
	Norvancomycin	Antimicrobial activity against Gram-positive bacteria (particularly MRSA and MRSE ^4^)	[92,93]
	Quartromicin (the complex of at least six antibiotics components A1, A2, A3, D1, D2, and D3)	Antiviral activity against herpes simplex virus type 1, influenza virus type A and human immunodeficiency virus	[94]
	UK-69753	Strong antimicrobial activity *in vitro* and *in vivo* against the swine Gram-negative anaerobic pathogen *Treponema hyodysenteriae*	[95,96]
	MM 47761 and MM 4972; MM 55266, and MM 55268	Antimicrobial activity against Gram-positive bacteria	[97,98]
	Eremomycin В	Antimicrobial activity against Gram-positive bacteria	[99,100,101]
	Orienticins A-D	Antimicrobial activity against *S. aureus* (including MRSA)	[102]
	Сhloroorienticins A-E	Antimicrobial activity against *S. aureus* (including MRSA)	[103]
	LY264826	Antimicrobial activity against Gram-positive bacteria (including MRSA)	[104]
	ECO-0501	Strong antimicrobial activity against Gram-positive bacteria (including MRSA and VRE)	[28]
*A. palatopharyngis*	Antibiotic biosynthetic genes were identified		[27]
*A. regifaucium*	Kigamicins A-E	Antimicrobial activity against Gram-positive bacteria (including MRSA); Kigamicin D is an anticancer agent	[48,105,106,107]
*A. rifamycinica*	Tetracenomycin Х	Moderate antimicrobial activity against Gram-positive organisms (including resistant strains); Activity against certain tumor cell lines	[108,109]
*A. roodepoortensis*	Antibiotic biosynthetic genes were identified; Antimicrobial activity against Gram-positive (particularly mycobacteria) and Gram-negative bacteria		[24]
*A. rubida*	Antibiotic biosynthetic genes were identified		[27]
*A. saalfeldensis*	Saalfelduracin	Strong antimicrobial activity against drug-resistant Gram-positive bacteria	[110]
*A. speibonae*	Antibiotic biosynthetic genes were identified; Antimicrobial activity against Gram-positive bacteria (particularly mycobacteria)		[24]
*A. speibonae*	Echinosporin 7-deoxyechinosporin	Antifungal activity against root-rot pathogens of the *Panax notoginseng*	[111]
*A. sulphurea*	Epoxyquinomicins A-D	Epoxyquinomicins A and B exhibit antimicrobial activity against Gram-positive bacteria; Epoxyquinomicins C and D exhibit almost no antimicrobial activity and no cytotoxicity; All these antibiotics showed improvement of collagen induced arthritis *in vivo*	[112,113]
	Azicemicins A and B	Moderate antimicrobial activity against Gram-positive bacteria (particularly mycobacteria)	[114,115]
	**Chelocardin** (Cetocycline)	Antimicrobial activity against Gram-positive and Gram-negative (including tetracycline-resistant pathogens and MDR pathogens)	[116,117,118]
*A. taiwanensis*	Antibiotic biosynthetic genes were identified		[27]
*A. thermoflava*	Antibiotic biosynthetic genes were identified		[27]
	1-methoxy-3-methyl-8-hydroxy-anthraquinone	Antibiotic biosynthetic genes were identified Anticancer activity against lung cancer and lymphoblastic leukemia cells	[119]
*A. tolypomycina*	Tolypomycin	Strong antimicrobial activities against Gram-positive bacteria	[65,120]
*A. tucumanensis*	Antibiotic biosynthetic genes were identified		[27]
*A. umgeniensis*	Eremomycin В	Antimicrobial activity against Gram-positive bacteria	[121]
*A. vancoresmycina*	Vancoresmycin	Antimicrobial activity against Gram-positive bacteria (including resistant strains)	[122,123]
*А. xylanica*	Antibiotic biosynthetic genes were identified		[27]

Strong antimicrobial activity—MIC ≤ 1 µg/mL, moderate—MIC 1–16 µg/mL, weak—MIC ≥ 16 µg/mL. ^1^ MRSA—methicillin-resistant *S. aureus*; ^2^ MDR—multiple drug resistance; ^3^ VRE—vancomycin-resistant enterococci; ^4^ MRSE—methicillin-resistant *S. epidermidis*; * Nomenclatural status: not validly published.

**Table 3:** Rifamycins and related metabolites produced by actinobacteria of the genus *Amycolatopsis*.

Rifamycin Metabolites	Possible Precursor	Properties	References
Proansamycin X	The first hypothetical macrocyclic intermediate of rifamycin biosynthesis has never been isolated and identified		[83,169]
Protorifamycin I (8-deoxyansamycins W)	Proansamycin X	No activity against Gram-positive bacteria or Gram-negative bacteria	[170]
modified protorifamycins (derived from protorifamycin I) and defective rifamycins (8-deoxyrifamycins)	Protorifamycin I	No antibiotic activity	[171,172]
Rifamycin W	Proansamycin X	No activity against Gram-positive bacteria or Gram-negative bacteria	[173]
Rifamycin Z	Rifamycin W	No activity against Gram-positive bacteria or Gram-negative bacteria	[174]
31-Homorifamycin W	Rifamycin W	No significant antibacterial, antifungal, or antiviral activity	[81]
Rifamycin SV	Rifamycin W	Strong activity against Gram-positive bacteria (particularly mycobacteria)	[175,176]
Rifamycin S	Rifamycin SV	Strong activity against Gram-positive bacteria (particularly mycobacteria)	[175]
Rifamycin R	Rifamycin S	Strong activity against Gram-positive bacteria (particularly mycobacteria)	[177]
Rifamycin G	Rifamycin S	Activity against *M. tuberculosis*	[178]
Rifamycin Y	Rifamycin B	Antibiotically inactive	[179,180]
Rifamycin YO, YS, Isorifamycin Y	Rifamycin Y	Antibiotically inactive	[179]
Protorifamycin B, 34a-deoxy-rifamycin W, Rifamycin W-28-desmethyl-28-carboxy, Rifamycin W-hemiacetal	Rifamycin W	No data	[181]
Rifamycin O	Rifamycin L	Activity against *M.abscessus*	[164,182,183]
Thiazorifamycins: Rifamycin Q, Rifamycin P, Rifamycin Verde	Rifamycin S	No data	[184]
Rifamycin L	Rifamycin S	Good antimicrobial activity against Gram-positive and Gram-negative bacteria	[164,185]
Rifamycin В	Rifamycin S	Activity against Gram-positive bacteria (particularly mycobacteria)	[79,164]
27-Demethoxy-27-hydroxyrifamycin derivatives	Rifamycin SV	Activity against several Gram-negative bacteria	[186]
3-Hydroxyrifamycin S and further novel ansamycins S, G and W type	Rifamycin S and W, respectively	Ansamycins W type are devoid of any biological activity. Other ansamacins exhibit activity against Gram-positive and Gram-negative bacteria	[187]
Rifamorpholines А-Е	Rifamycin S	Rifamorpholines B and D exhibit antimicrobial activity against methicillin-resistant *S. aureus* (MRSA)	[13]

Strong antimicrobial activity—MIC ≤ 1 µg/mL, moderate—MIC 1–16 µg/mL, weak—MIC ≥16 µg/mL.

## Supplementary Materials

The following are available online at https://www.mdpi.com/article/10.3390/antibiotics10101254/s1, Figure S1: Biosynthetic pathway of vancomycin [132,133]. Figure S2: (A) Biosynthetic pathway of rifamycin B [163,164]. (B) Chemical structure of rifampicin.
