# Supplementary figures and images for: Looking Back to Amycolatopsis: History of the Antibiotic Discovery and Future Prospects
Source: Antibiotics (Basel). 2021 Oct 15;10(10):1254. doi: 10.3390/antibiotics10101254 (PMC8532670; doi:10.3390/antibiotics10101254)

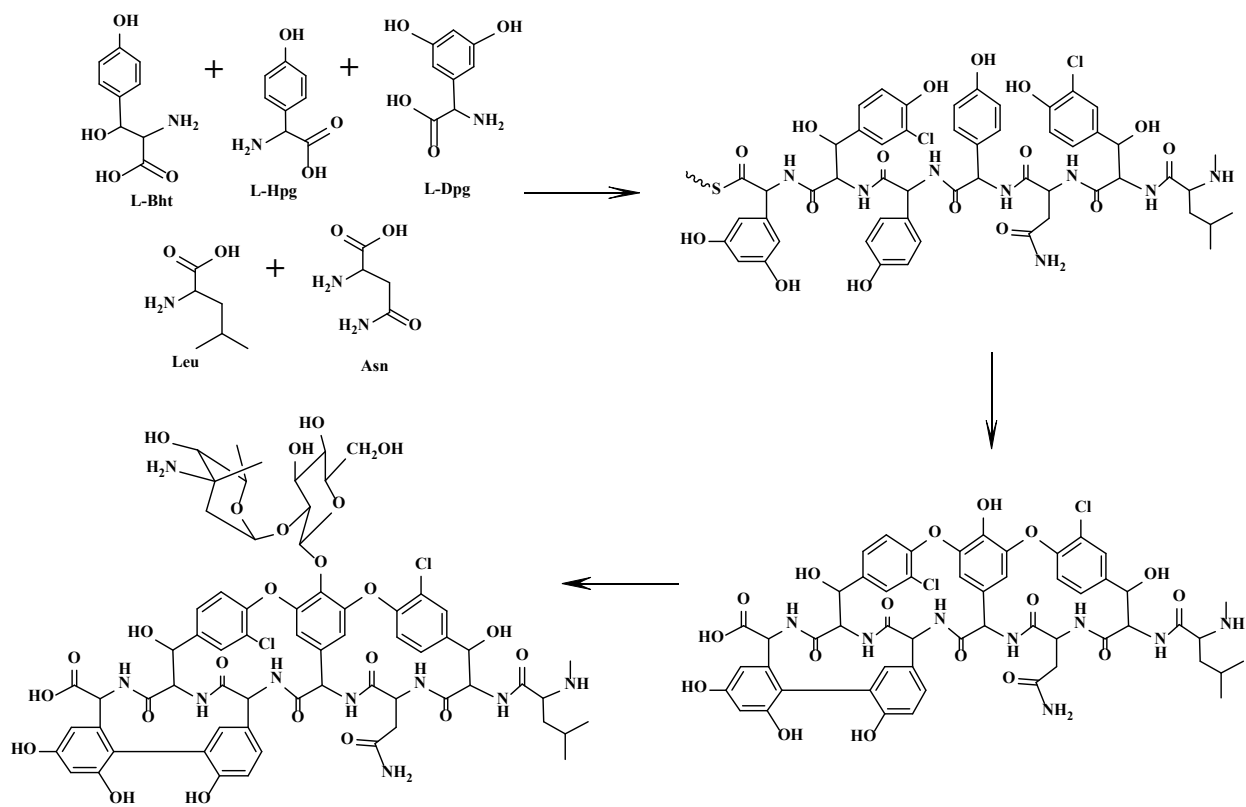

Figure S1. Biosynthetic pathway of vancomycin [132–134].

Supplement: Supplementary file 1 [file antibiotics-10-01254-s001.zip › antibiotics-1362528-supplementary.pdf]
